# Joint embedding: A scalable alignment to compare individuals in a connectivity space

**DOI:** 10.1016/j.neuroimage.2020.117232

**Published:** 2020-11-15

**Authors:** Karl-Heinz Nenning, Ting Xu, Ernst Schwartz, Jesus Arroyo, Adelheid Woehrer, Alexandre R. Franco, Joshua T. Vogelstein, Daniel S. Margulies, Hesheng Liu, Jonathan Smallwood, Michael P. Milham, Georg Langs

**Affiliations:** a Computational Imaging Research Lab, Department of Biomedical Imaging and Image-guided Therapy, Medical University of Vienna, Vienna, Austria; b Center for the Developing Brain, Child Mind Institute, New York, NY, USA; c Center for Imaging Science, Johns Hopkins University, Baltimore, MD, USA; d Division of Neuropathology and Neurochemistry, Department of Neurology, Medical University of Vienna, Vienna, Austria; e Center for Biomedical Imaging and Neuromodulation, Nathan Kline Institute, Orangeburg, NY, USA; f Department of Psychiatry, NYU Langone School of Medicine, New York, NY, USA; g Department of Biomedical Engineering, Institute for Computational Medicine, Kavli Neuroscience Discovery Institute, Johns Hopkins University, Baltimore, MD, USA; h Centre National de la Recherche Scientifique, Frontlab, Institut du Cerveau et de la Moelle Epinière, Paris, France; i A.A. Martinos Center for Biomedical Imaging, Massachusetts General Hospital, Harvard Medical School, Boston, MA, USA; j Department of Psychology, Queen’s University, Kingston, Ontario, Canada; k Computer Science and Artificial Intelligence Laboratory, Massachusetts Institute of Technology, Cambridge, MA, USA

**Keywords:** Functional alignment, Joint embedding, Functional gradient, Lifespan, Common space, Individual differences

## Abstract

A common coordinate space enabling comparison across individuals is vital to understanding human brain organization and individual differences. By leveraging dimensionality reduction algorithms, high-dimensional fMRI data can be represented in a low-dimensional space to characterize individual features. Such a representative space encodes the functional architecture of individuals and enables the observation of functional changes across time. However, determining comparable functional features across individuals in resting-state fMRI in a way that simultaneously preserves individual-specific connectivity structure can be challenging. In this work we propose scalable joint embedding to simultaneously embed multiple individual brain connectomes within a common space that allows individual representations across datasets to be aligned. Using Human Connectome Project data, we evaluated the joint embedding approach by comparing it to the previously established orthonormal alignment model. Alignment using joint embedding substantially increased the similarity of functional representations across individuals while simultaneously capturing their distinct profiles, allowing individuals to be more discriminable from each other. Additionally, we demonstrated that the common space established using resting-state fMRI provides a better overlap of task-activation across participants. Finally, in a more challenging scenario - alignment across a lifespan cohort aged from 6 to 85 - joint embedding provided a better prediction of age (r2 = 0.65) than the prior alignment model. It facilitated the characterization of functional trajectories across lifespan. Overall, these analyses establish that joint embedding can simultaneously capture individual neural representations in a common connectivity space aligning functional data across participants and populations and preserve individual specificity.

## 1 Introduction

Understanding the functional organization of the brain is a fundamental goal in neuroscience. Manifold learning has been shown to be a viable tool for representing functional connectivity structure, in a manner that is uncoupled from anatomy (Haak et al., 2018; Langs et al., 2016, 2014; Margulies et al., 2016; Paquola et al., 2019). It assumes that the underlying structure of relationships lies embedded on a manifold in a higher-dimensional space that can be captured by a low-dimensional representation. A growing number of studies have shown that the components of diffusion map embedded resting-state fMRI (rs-fMRI) data can encode biologically meaningful structure. Such components, referred to as functional connectivity gradients, can represent the hierarchy of information processing from primary to transmodal regions (Margulies et al., 2016). The topographical pattern of the components reflects the cognitive landscape from perception to the abstract function which aligns with cortical myelination (Huntenburg et al., 2017). Similar observations were obtained for the hippocampus and cerebellum in humans (de Wael et al., 2018; Guell et al., 2018), as well as in nonhuman primates including macaque and marmoset (Buckner and Margulies, 2019; Xu et al., 2019).

Characterizing individual-specific diffusion embedding maps in a functional space facilitates the establishment of correspondences across individuals as well as the comparison between populations (de Wael et al., 2019; Langs et al., 2016, 2011). For example, embedding representations were used to reveal an atypical functional hierarchy in autism (Hong et al., 2019), and to uncouple function from structure to capture functional correspondences across controls and brain tumor patients (Langs et al., 2014). Recently, embedding coefficients of shared functional components were used to establish features for functional cortical alignment across healthy participants (Nenning et al., 2017) and to detect common functional characteristics to guide cross-species alignment between human and macaque (Xu et al., 2019).

While embeddings have proven useful in characterizing brain architecture and distinguishing between populations, matching individual functional gradient representations across participants remains a challenge. In particular, the individual-specific functional structure can be variable and artifacts in the recorded connectivity structure can impact the properties of the embedding space which may make matching of corresponding systems across people difficult. Spectral embedding methods do not directly provide comparable mappings since the eigenvalue decomposition can result in coefficient sign flips, rotations, and differences in the ordering of the components (Langs et al., 2016, 2011). As such, finding embedding representations of multiple graphs simultaneously is the key to overcome these difficulties (Arroyo et al., 2019; Ham et al., 2005, 2003; Levin et al., 2017; Lombaert et al., 2013a,b; Wang et al., 2019).

Recently, we have developed a joint embedding approach that establishes matched embedding representations and we successfully applied this method to align functional connectivity structure across species (Xu et al., 2019). While computational feasibility was not a concern in the initial framework focused on group-level representations of connectivity for different species, it is a concern for its broader application. Here, we propose a scalable version of the joint embedding approach to overcome the challenge of matching individual embeddings for vertex-wise inference in large datasets. We aim to establish a common connectivity space capturing functional gradients matched across individuals. It is worth noting that the representative gradients are flexible to be extended as functional features for cortical alignment (Nenning et al., 2017). However, unlike cortical alignment, here we focus on the matching algorithm in a common connectivity space as the goal of the present work is to identify representative functional connectivity gradients across individuals, providing a robust solution to facilitate inter-individual analysis.

In this study, we evaluate the scalable joint embedding approach using both task and resting-state data from the Human Connectome Project (HCP) and lifespan data from the enhanced Nathan Kline Institute-Rockland Sample (NKI-RS). We compare the joint embedding framework to a previously established orthonormal alignment approach, which calculates an embedding for each participant individually with subsequent matching across the cohort (Langs et al., 2015, 2014, 2011). We show that joint embedding provides a better mapping of individuals into a common space, increasing within and between individual similarity, and improving the ability to identify individuals. Additionally, we demonstrate that it is suitable for age prediction and thus facilitates analysis of embedding trajectories across the human lifespan.

## 2 Material and methods

### 2.1 Datasets

#### 2.1.1 HCP sample

We used the 100 unrelated subjects (54F/46M, age = 29 ± 3.7 years) from the S500 young adult data release (Glasser et al., 2013; Van Essen et al., 2012). We split the data into two subsets and used the rs-fMRI data to construct the functional common space per subset and to allow cross-validation across subsets. Two resting-state scans (REST1_LR and REST2_LR) for each individual were included in the present study. Additionally, we used task fMRI data to quantify activation overlap in the common space created from resting-state data. Seven tasks with multiple experimental conditions were included (i.e. language, motor, working memory, gambling, social cognition, relational, and emotional processing).

The HCP minimally preprocessed data was further preprocessed (Wang et al., 2015). First, we resampled the data (i.e. MSMSulc registered) to fsaverage6 (41k vertices per hemisphere) using the resampling deformation sphere from the HCP Pipeline (https://github.com/Washington-University/HCPpipelines/tree/master/global/templates/standard_mesh_atlases/resample_fsaverage). Additional preprocessing for resting-state data includes detrending, bandpass filtering (0.01–0.08 Hz), head motion regression, global signal regression, and smoothing (FWHM = 6 mm) on the surface (i.e. fsaverage6). Finally, we downsampled the resting-state fMRI data and task activation maps on the FreeSurfer fsaverage4 template (2562 vertices per hemisphere). We selected fsaverage4 in our main analyses for two reasons. First, as our goal is to construct the functional space, instead of using parcellations, fsaverage4 has no prior assumption for the initial nodes, or assumption of vertex-to-vertex symmetry across hemisphere. Second, *fsaverage4* provides a relatively coarse but representative resolution to help with the computational costs. To confirm the reproducibility on a higher resolution surface, we also tested our algorithm with a higher resolution surface (i.e. 10k_fs_LR) on one subset of the HCP data using the other subset for constructing the common space reference (details see Figure S6 and the result Section 3.1.2).

#### 2.1.2 NKI-RS lifespan

The NKI dataset was obtained from the publicly shared enhanced Nathan Kline Institute-Rockland Sample data repository (http://fcon_1000.projects.nitrc.org/indi/enhanced/). In the current study, we selected 312 participants (214 female, age: 6-85 years, 42.2 ± 22.4 years) who have no diagnosis of any mental or neurological disorders, and passed quality control of a head motion criteria (mean framewise displacement < 0.25mm). For each individual, a high-resolution T1-weighted scan (1 mm isotropic resolution, TR = 1900 ms, TE = 2.52 ms, Flip angle = 9°) and a 10-min rs-fMRI acquisition (3 mm isotropic resolution, TR = 645 ms, TE = 30 ms, Flip angle = 60°) were included in the current study. The details of the dataset and sequences are described elsewhere (Nooner et al., 2012).

The NKI dataset was preprocessed using the Connectome Computational System (CCS: https://github.com/zuoxinian/CCS) (Xu et al., 2015). Briefly, the structural MRI preprocessing included spatial denoising (CAT12), brain extraction, segmentation, and surface reconstruction. The deformation sphere from the native surface to fsaverage space was calculated in FreeSurfer. We then generated the standard surface meshes, for which each node has one-to-one correspondence with a node on the high-resolution fsaverage surface in native space. The functional image preprocessing included discarding the first five timepoints, compressing temporal spikes (AFNI 3dDespike), slice timing correction, motion correction, 4D global mean intensity normalization, nuisance regression (Friston's 24 model, cerebrospinal fluid and white matter), linear and quadratic detrends, as well as band-pass filtering (0.01–0.1 Hz). The preprocessed data were then registered to the anatomical space using boundary-based registration and projected to the high-resolution surface (i.e. fsaverage) in native space. The rs-fMRI data were then spatially smoothed on the fsaverage surface (FWHM = 6 mm). Finally, similarly to the HCP dataset, the data were downsampled to fsaverage4 surface for the following analyses.

### 2.2 Joint embedding

**Fig. 1:**
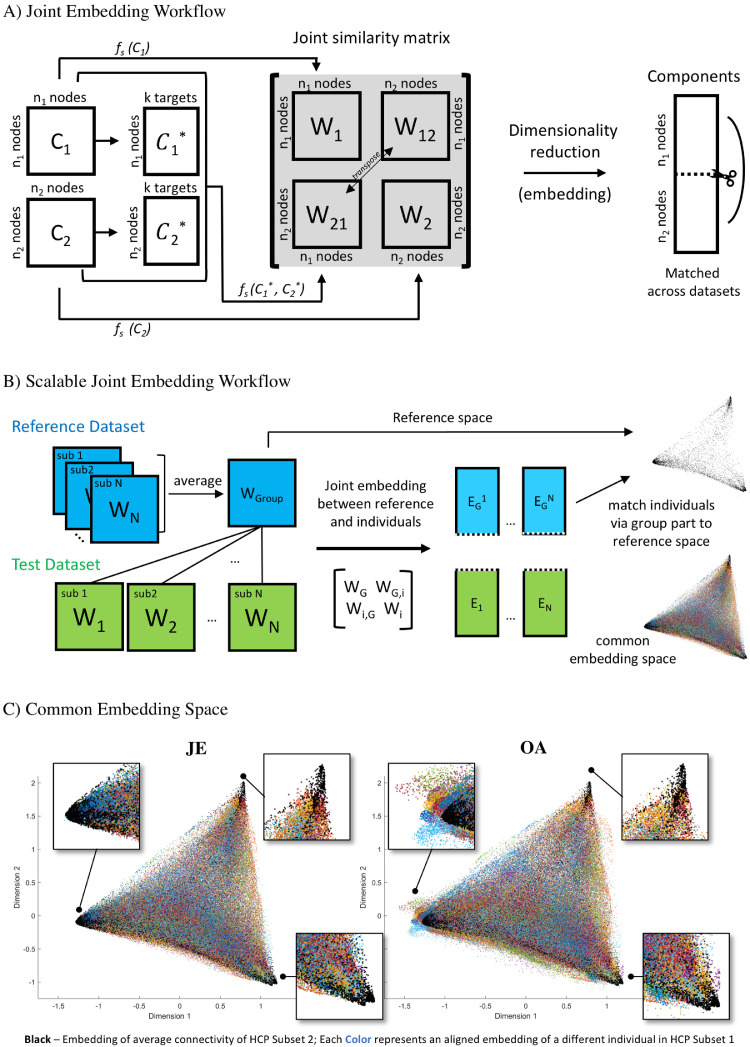
The joint embedding workflow. A) The rationale behind joint embedding. C denotes a connectivity matrix with n nodes, and f(C) the kernel to compute a similarity matrix W. B) The scalable joint embedding alignment across multiple subjects. C) Illustration of the common space established with joint embedding (JE), and alignment of individual embeddings (OA).

A growing number of recent studies used the diffusion map technique to quantify embeddings of functional connectivity (Langs et al., 2016; Margulies et al., 2016; Nenning et al., 2017; Paquola et al., 2019). In brief, diffusion maps determine a non-linear mapping of a dataset to a low-dimensional representational space that captures the local and global structure of the data. The embedding is based on the eigenvalue decomposition of a transition probability matrix which quantifies the similarity structure within a dataset (Coifman and Lafon, 2006). In joint embedding, the spectral embedding decomposition is placed on a joint similarity matrix across datasets so that the resultant components are matched and comparable (Ham et al., 2005, 2003; Lombaert et al., 2013a,b; Xu et al., 2019). As such, joint embedding acts as a regularization of the variability across datasets, facilitating alignment and at the same time being faithful to individual traits from each dataset. For simplicity we describe the joint embedding of two datasets, but the method can be extended to comprise more than two datasets following the same rationale. An illustration of the joint embedding workflow is given in Fig. 1A.

Consider two symmetric connectivity matrices *C_1_* (*n_1_* nodes) and *C_2_* (*n_2_* nodes). We construct a joint similarity matrix by concatenating within- and across-dataset similarity matrices *W_joint_* = [*W_1_ W_12_; W_21_ W_2_*]. Where the diagonal block matrices *W_1_* and *W_2_* are the cosine similarity of connectivity profiles (Margulies et al., 2016). To obtain a coupling off-diagonal similarity matrix between two datasets, for each dataset, we first calculate node-wise connectivity to *k* targets that are comparable across datasets (C_1_*, C_2_*). Subsequently the off-diagonal coupling matrices *W_12_* and *W_21_* are established via cosine similarity of C_1_* and C_2_*. A single joint similarity matrix *W_joint_* is then created by concatenating within-dataset similarities and cross-dataset coupling matrices. Placing the diffusion map embedding on *W_joint_* results in a set of comparable embedding components *E*. For each component the first *n_1_* nodes represent the latent position of *C_1_* and the following *n_2_* nodes for *C_2_* in the common connectivity space.

Following prior studies (Margulies et al., 2016; Paquola et al., 2019), the functional connectivity matrices are calculated with Pearson's correlation and thresholded at the top 10% strongest connectivity at each vertex. In the current study, the nodes are the vertices of the anatomically aligned fsaverage4 surfaces for all participants (*n_1_* = *n_2_* = 4680 vertices included in the cortex mask), and each anatomically aligned vertex was used as a target node. We also evaluated the impact of the number of k targets on the alignment (*k* = 5–100% of all vertices) by comparing the similarity of component profiles within individuals (Fig. S1).

### 2.3 Common space and alignment

Joint embedding implicitly results in comparable components, but due to the growing number of individuals in fMRI datasets it is usually unfeasible to yield a large single joint matrix and perform joint embedding. To address this problem, we propose to perform joint embedding between the connectivity matrices of the group average and each individual in a cohort individually (Fig. 1B). This will chain together the individual participants via the group average, and the inclusion of the group average matrix will modulate the participant-specific embeddings to lie in a common space. Specifically, the joint similarity matrix is constructed as *W_joint_* = [*W_G_ W_iG_; W_Gi_ W_i_*], where *W_G_* is the similarity matrix of the group average, *W_i_* the similarity matrix of an individual and *W_Gi_* the coupling matrix between group average and individual with *W_iG_* as its transpose. Thus, the obtained set of comparable embedding components *E* consists of the group embedding *E_G_^i^* and the aligned individual embedding *E_i_*. The final order and direction of *E_i_* across individuals were determined by a procrustes transformation to account for order and sign flips. Consistent with the previous studies, we determined the number of components as the inflection point of eigenvalues (lambdas) on the scree plot, resulting in the first 25 components used as an embedding profile in this study. The aligned components represent shared functional gradients across individuals and were used to construct the common space. We illustrated the first and second dimensions of the common space in Fig. 1C and demonstrated how well individual components from JE and OA were aligned with the reference in the common space. Note that the correspondence between anatomy space and the functional common space is preserved in JE; each node (e.g. vertex or voxel depending on the input data type) in the anatomy space corresponds to each node in the functional space. As such, we were able to project the components back to the anatomy space and localize the embedding component scores at each vertex of the cortical surface.

It is worth noting that the alignment of functional gradients in the common space (Fig. 1C) is feasible using other surfaces or volume data without node-to-node anatomy-based pre-alignment with the reference (Langs et al., 2010, 2014). This advantage enables identifying functional areas in the case that the anatomical alignment is difficult to achieve (e.g. tumor patients) (Langs et al., 2014). In Fig. S2, we illustrated how components from individual volume space were matched directly to the reference surface in the common space. Additionally, we also measured the similarity of components on a higher resolution surface mesh (i.e. fsaverage5, 10k per hemisphere) and found highly similar spatial profiles of resulting gradients with the coarse surface used in the main analyses (*r* > 0.9 across 25 components) (Fig. S3).

### 2.4 Evaluation

To evaluate and confirm reproducibility, we split the HCP dataset into 2 subsets with 50 participants each (subset 1: 25 females, subset 2: 23 females). To ensure the independence of the group reference, we used the group average of subset 1 as the reference and evaluated the alignment of individuals in subset 2, and vice versa. We compared joint embedding (JE) to the previously established approach based on orthonormal alignment (OA) of individual embeddings (Langs et al., 2015, 2014, 2011) with the following procedure.

1) *Similarity to the reference in the common space.* For each vertex, we evaluated the similarity (Pearson's r) of its embedding profile between each individual and the reference in the common space. An improved alignment achieves higher average similarity and lower standard deviation in similarity across individuals.
2) *Similarity within and between individuals.* We evaluated within individual similarity of embedding profiles between the two repeated resting scans and pairwise similarities between individuals with Pearson's correlation. We quantified the average and their standard deviation at the group level, where an improved alignment provides higher average similarity and lower standard deviation within individuals.
3) *Ability to identify individuals.* Optimal inter-individual alignment increases the overall similarity of features across individuals while simultaneously maintaining individual differences. Here, we calculated discriminability (Bridgeford et al., 2019), a multivariate nonparametric statistic to quantify the extent to which an individual can be identified, for each embedding component. Specifically, we quantified the fraction where the within-individual distances are lower than the cross-individual ones. A high discriminability indicates a better individual identification, and a group embedding that more faithfully captures individual differences.
4) *Task activation overlap in common space.* We evaluated the generalization of joint embedding by quantifying the task activation overlap across individuals in the common space established based on resting-state fMRI. This was carried out by testing if the task-active vertices across individuals are more densely distributed in the joint-embedding space. We used a leave-one-out prediction scheme to evaluate the task activation overlap across subjects. Specifically, for each vertex of a given individual, we identified the nearest neighbor from each other individual (N-1) in the common space and mapped the corresponding z-scores. Next, we quantified the similarity of task activation by calculating the Pearson correlation of the z-scores from a given individual and the averaged z-scores from the N-1 individuals. A paired t-test was conducted to test the difference between JE and OA for each of the 50 task contrasts. In addition, we further calculated pairwise Dice coefficients of thresholded activation maps across all the individuals for various thresholds. A higher correlation and dice coefficient determine that task-active vertices were surrounded by similar task-active vertices from other individuals in the functional space without anatomy-spatial constraints. Improved alignment should result in a higher correlation and dice coefficients, since it brings functionally similar regions, indicated by the task activation, into correspondence.

### 2.5 Alignment of lifespan sample capturing shared transition of the connectome

To test whether JE can improve alignment in a lifespan dataset with a large age span compared to OA, we performed support vector regression (SVR) to predict age and compared the performances of JE and OA. We used a linear kernel and a 20-fold cross validation. The vertex-wise embedding coefficient profiles, subtracted by the coefficients of the HCP reference, were used as features for the SVR model. Of note, the prediction was performed in the NKI-lifespan sample while the reference was generated based on the HCP dataset to ensure independence in the prediction. The prediction results were aggregated from all 20 folds to obtain the final mean average error (MAE) and r-squared values for JE and OA, respectively. To characterize the age-related changes of embedding coefficients and their ranges across the lifespan, we also used a general linear model including linear and quadratic terms of age effects.

### 2.6 Data and code availability

All data are publicly available at the Human Connectome Project (HCP, https://www.humanconnectome.org) and the enhanced Nathan Kline Institute-Rockland Sample data repository (NKI-RS, http://fcon_1000.projects.nitrc.org/indi/enhanced/). The preprocessing code for NKI data is available at https://github.com/zuoxinian/CCS. The Discriminability code is available at https://github.com/neurodata/discriminability. The JE code can be found at https://github.com/khne/JointEmbedding.

## 3 Results

### 3.1 HCP Evaluation

#### 3.1.1 Similarity to common space

**Fig. 2:**
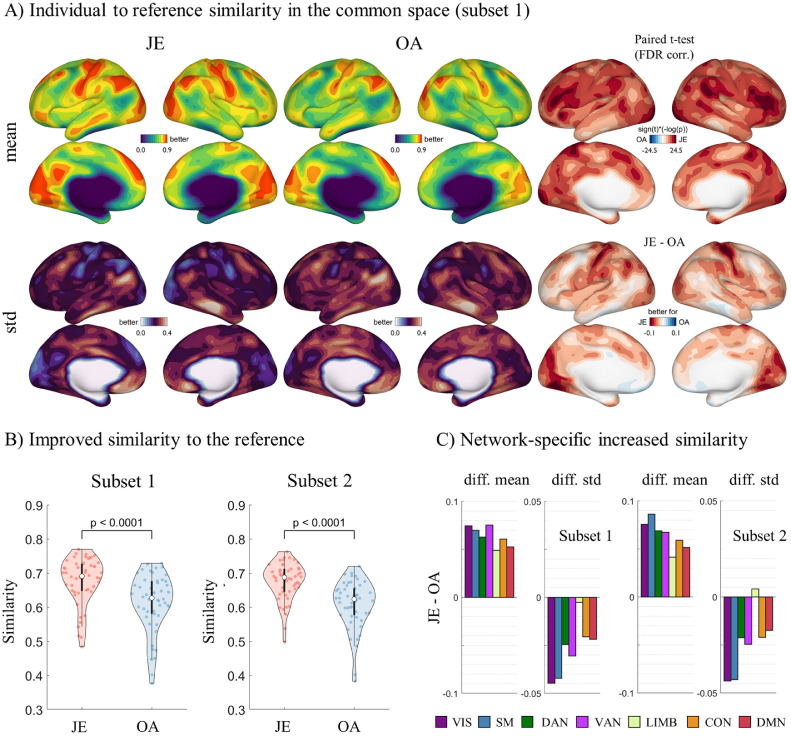
Average similarity of individual embedding component profiles to the reference. A) Notable cortical patterns of higher similarity and reduced variation are observed in JE compared to OA (see subset 2 results in Fig. S2). B) A paired *t*-test for individuals reveals a significant improvement of JE over OA for both subsets (subset1: t(49)=22.88, *p <* 0.0001; subset2: t(49)=24.54, *p <* 0.0001). C) Similarity of embedding component profiles to the reference at network level shows an increased similarity and reduced variance, particularly in somatosensory and visual networks.

Overall, the individual-specific similarity to the reference in the common space was significantly higher for JE compared to OA (Figs. 2A and Fig. S4). Results were consistent for both subsets (paired t-test, subset 1: t(49) = 22.88, *p* < 0.0001; subset 2: t(49) = 24.54, *p* < 0.0001) (Fig. 2B). Notably, JE resulted in a higher similarity for all resting-state networks, particularly of visual, somatomotor and attention networks (Fig. 2C). Additionally for JE, all networks except the limbic (in subset 2 only) showed a reduced standard deviation of similarities, with the most reduction observed in the visual and somatomotor network.

#### 3.1.2 Within, between individual similarity and discriminability

**Fig. 3:**
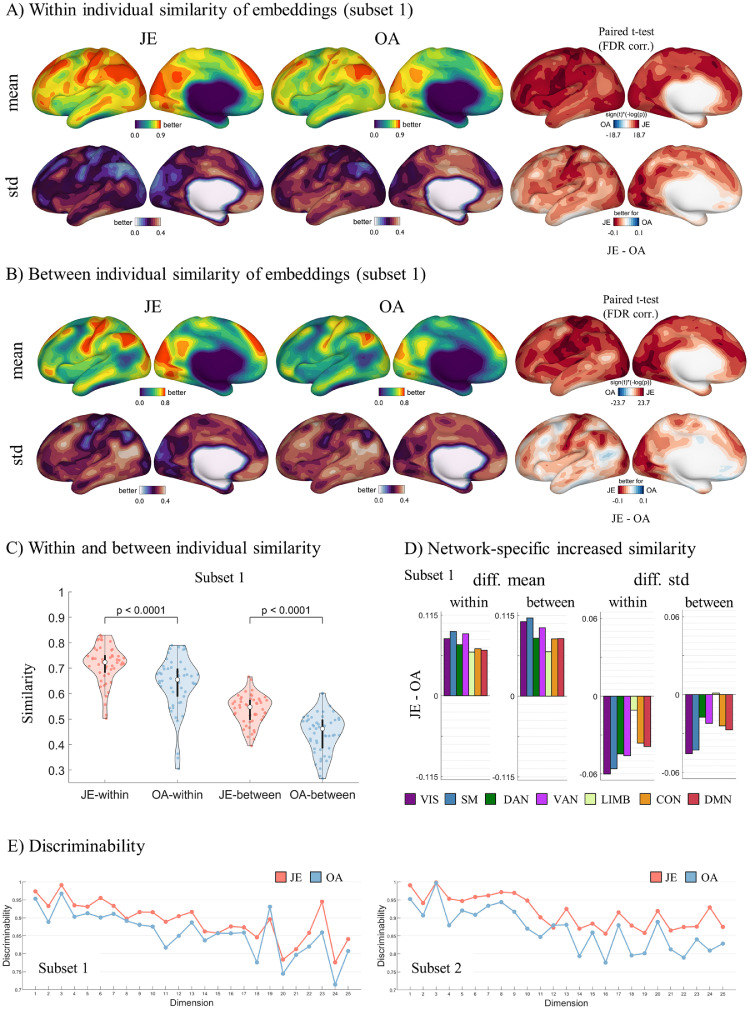
Average within and between individual similarity of embedding component profiles in the common space (subset 1). A) JE shows increased within individual similarity and reduced variation compared to OA. B) JE shows increased between individual similarity and reduced variation compared to OA. C) A paired *t*-test reveals significantly higher within and between individuals similarity for JE compared to OA (within: t(49)=13.85, *p <* 0.0001; between: t(49) = 25.61, *p <* 0.0001). D) The individual variance for JE is lower than for OA across networks, particularly for somatosensory and visual networks. E) JE components show a higher discriminability than OA, revealing that the individual pattern from JE is more identifiable as compared to OA components.

Within and between individual similarity were both significantly higher for JE compared to OA (paired *t*-test, subset 1: within: t(49) = 13.85, *p <* 0.0001; between: t(49) = 25.61, *p <* 0.0001) (Fig. 3), and consistent for both subsets (paired *t*-test, subset 2: within: t(49) = 14.26, *p <* 0.0001; between: t(49) = 29.63, *p <* 0.0001) (Fig. S5). At the network level, JE resulted in a higher within and between individual similarity across all networks, while a reduced standard deviation for all networks except the limbic network (Fig. 3D for subset 1 and Fig. S5 for subset 2). Notably, JE showed a particularly reduced standard deviation of between-individual similarity in the primary systems including visual and somatomotor networks. Importantly, discriminabilities obtained for JE in both subsets were higher than OA, indicating a better ability to identify individuals using JE (Fig. 3E). We also tested JE and OA on a higher resolution surface (i.e. 10k_fs_LR), finding consistent results that JE yielded higher within, between individual similarity and greater discriminability (Fig. S6). Overall, these results suggested that JE can not only characterize the common features across individuals but simultaneously preserve the individual-specific connectivity traits to fingerprint their connectivity structure.

#### 3.1.3 Task activation overlap

**Fig. 4:**
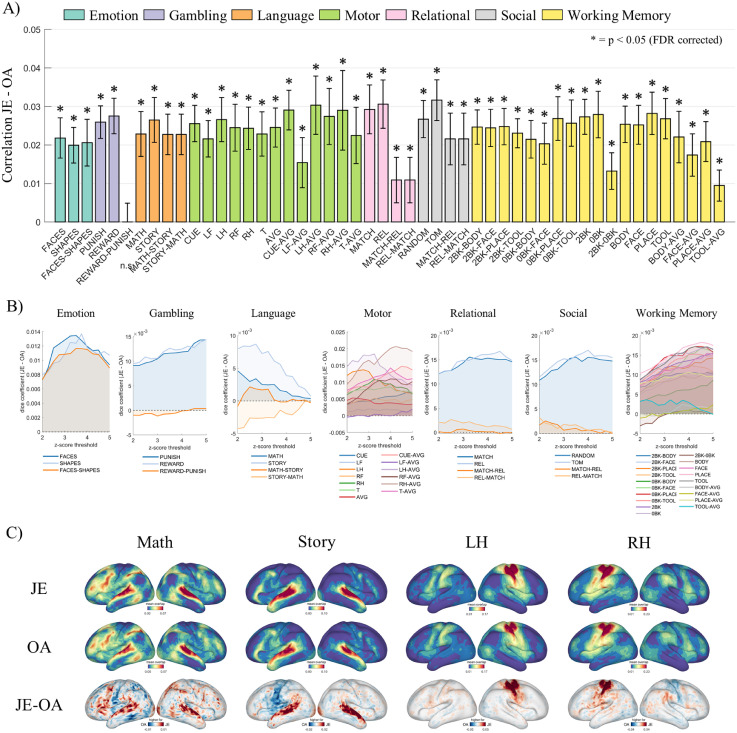
Higher overlap of task-activation across participants in the JE common space compared to OA for subset 1. A) For JE compared to OA, a significantly higher correlation between actual and predicted z-scores is observed in 49 of 50 task-contrasts (paired t-test for 50 individuals in subset, *p* < 0.05 FDR corrected). B) The averaged difference (JE-OA) of pairwise Dice coefficients between thresholded z-maps at various thresholds. C) The averaged thresholded task-activation maps (z-score > 3.1) show a higher overlap for JE in task-active regions.

For both subsets, JE achieved higher task activation overlap across individuals compared to OA (Fig. 4 for subset 1 and Fig. S7 for subset 2). There are 49/50 task conditions in subset 1 and subset 2 that showed a significantly higher correlation between actual and predicted activation for JE (paired t-test, *p <* 0.05 FDR corrected). The difference in correlation is shown in Fig. 4A, and corresponding correlation values between actual and predicted z-score maps for JE and OA are given in Fig. S8. Additionally, pairwise Dice coefficients revealed a better overlap for JE over various thresholds (Fig. 4B). The averaged thresholded task-activation maps (z-score > 3.1; *p <* 0.001, one tail) revealed condition specific patterns of task relevant regions where JE has a higher task activation overlap than OA (Fig. 4C). As a reference, the averaged thresholded task-activation at z-score > 3.1 for anatomical alignment is provided in Fig. S8C.

### 3.2 Lifespan analysis

**Fig. 5:**
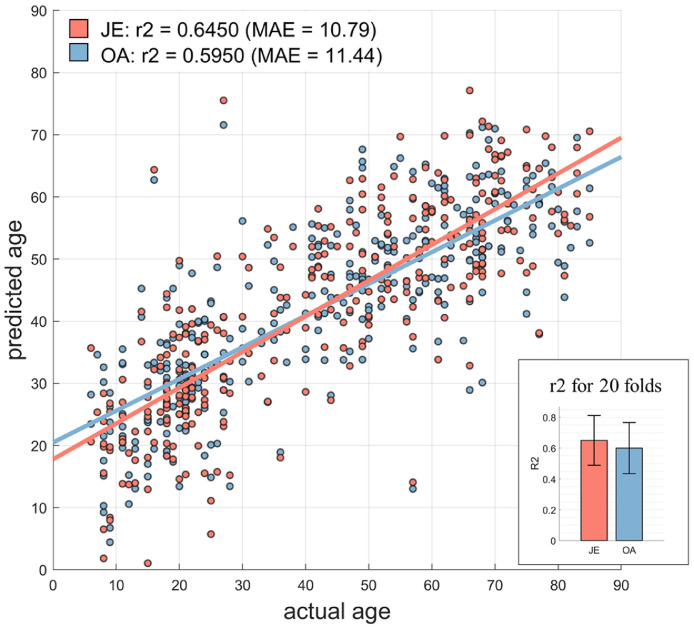
Embedding component profiles based on JE outperform OA for age prediction in the lifespan sample. On aggregate, age prediction from all 20 folds results in a lower MAE for JE (paired *t*-test, t(311) = −2.6871, *p =* 0.0076). Across all 20 folds, JE (mean r2 = 0.6505 ± 0.1617) showed higher r-square than OA (mean r2 = 0.6002 ± 0.1661) (paired *t*-test t(19) = 2.75, *p =* 0.0127).

As shown in Fig. 5, better age prediction performances were obtained with JE. Across all 20 folds, JE (mean r2 = 0.6505 ± 0.1617) achieved a better prediction performance compared OA (mean r2 = 0.6002 ± 0.1661, paired *t*-test t(19) = 2.75, *p* = 0.0127). Aggregated over all folds, JE achieved a mean absolute error (MAE) of 10.79 years and an r2 = 0.6450, compared to OA with MAE of 11.44 years and r2 = 0.5950, and a paired *t*-test for the age prediction error revealed a significant lower MAE for JE (t(311)=−2.6871, *p =* 0.0076). In addition, we also performed age prediction using PCA on the whole brain connectivity matrix to provide a prediction baseline. Similar with JE and OA, the first 25 PCA components were used in the SVR model. Across 20 folds, JE showed a relatively higher accuracy and lower MAE than PCA (r2 = 0.6258, MAE = 11.43, t(311)=−2.1626, *p =* 0.0313), while no significant difference was observed between PCA and OA (*p =* 0.9736). Fig. S9 shows the feature weights for the first 12 JE components, illustrating distinct spatial patterns contributing to the age prediction.

**Fig. 6:**
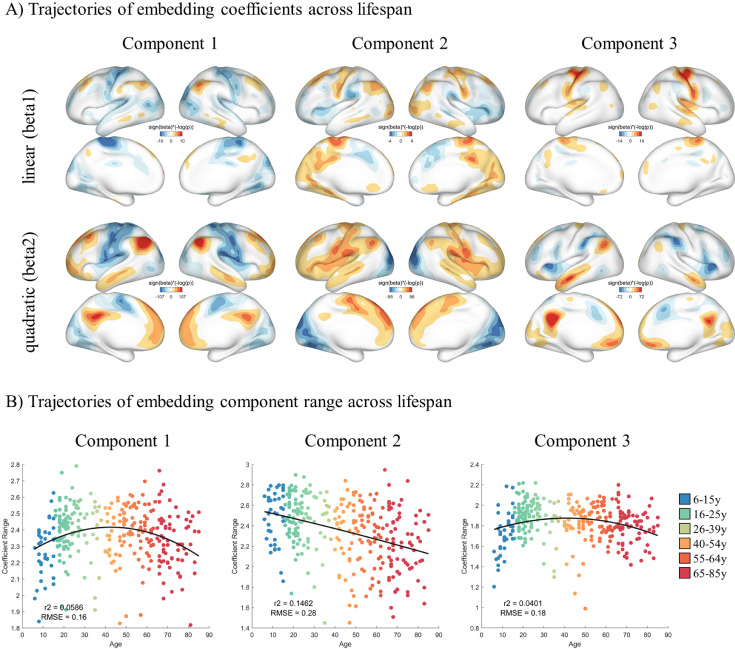
Trajectories of JE components across lifespan. A) Linear and quadratic trend of JE components across lifespan. B) Scatter plots of the range of the first three components across lifespan.

We further characterized the linear and quadratic changes of JE components across lifespan. Animation1 shows the estimated trajectory of functional connectivity embeddings along dimension 1 and 2 in the common space and their corresponding changes on the cortical surface based on the lifespan sample (6–85 age). Animation 2 illustrates the lifespan gradient trajectories along dimension 2 and 3. Fig. 6A shows the significant (FDR corrected) linear or quadratic relationships between individual embedding coefficients and age across the cortex. The spatial patterns of the significance maps were highly similar to their own embeddings maps (component 1: *r* = 0.90; component 2 *r* = 0.92; component 3 *r* = 0.85), indicating that the apex or nadir of embedding components in functional space situate at the regions that change the most across the lifespan. In addition, we applied the regression model to the range of the embedding components (the apex minus nadir) and revealed inverted U-shape trajectories for components 1 and 3, while a linear decline with age for component 2 (Fig. 6B).

**Animation 1.** Trajectories in the common space reveal changes in the functional connectivity structure across lifespan along components 1 and 2.

**Animation 2.** Trajectories in the common space reveal changes in the functional connectivity structure across lifespan along components 2 and 3.

## 4 Discussion

Determining comparable representations of individual connectomes is the key to being able to understand the relationship between patterns of neural organization across individuals and situations. In this work, we proposed a new approach - joint embedding (JE) - as a methodology to identify comparable maps across datasets and drive their alignment in a common functional space. We demonstrated that JE significantly improves individual matching compared to the established orthonormal alignment approach. The JE maps have a higher within and between individual similarity while simultaneously preserving high discriminability of individual representations to allow identifying individuals from the sample. In addition, the JE common space created using resting-state fMRI is generalizable to spatial maps of task activation, allowing overlapping regions of activity across individuals to be determined within the embedded space. Finally, we were able to use JE to characterize a lifespan sample with a large age span (6 to 85), facilitating age prediction with a reasonable degree of accuracy (r2 = 0.6450). These results establish that JE creates a common embedding space that can be used to understand common and unique features of functional neural activity from different individuals and in different situations.

### 4.1 JE improves individual similarity and discriminability

Compared to the previously established orthonormal alignment and consistently over both subsets, JE provides a higher individual-specific similarity to the reference common space, as well as an increased within and between individual similarity across all cortical networks. This was especially evident for somatosensory and visual networks, which have been shown to exhibit a low variability across individuals (Mueller et al., 2013). Similar to the findings on fsaverage4, JE improves matching of embedding components and enables establishing a more coherent connectivity space than OA for data on the 10k fs_LR surface (Fig. S6). This suggests that the common space provided by JE encodes individual-specific connectivity structure more accurately than orthonormal alignment.

One challenge in inter-individual alignment is to preserve the individual-specific features while optimizing the similarity across individuals. An optimal inter-individual alignment should allow for similarity between individuals while simultaneously preserving unique features of the individual. Our analysis suggests that JE not only improved within and between individual similarities, more importantly, it also significantly increased the ability to identify individuals based on neural features (i.e. fingerprinting) as it revealed a higher discriminability (Bridgeford et al., 2019). This establishes that the common space provided by JE improves the possibility for both inter-individual alignment while preserving the information necessary for the separation of different participants. With increasing dimensionality, the separation between components becomes less pronounced as the coefficients capture a similar amount of variation in the data for both JE and OA alignments. However, JE consistently provided better discriminability across components, supporting results of a more accurate common space particularly in low variance components. Noteworthy, in early high variance components, a drop in discriminability is observed at the second embedding dimension which encodes the visual - somatosensory connectivity axis, exhibiting low variability across individuals (Mueller et al., 2013). This may reflect the fact that neural activity in perceptual and motor systems may be relatively preserved across participants. Overall, our analysis suggests that JE creates a common embedding space which efficiently balances the need to identify neural features that are common to an individual, while maintaining unique features associated with individual neural function that allows discrimination of different individuals.

### 4.2 Increased task activation overlap

In the JE common space established with task-free resting-state data, vertex-wise neighborhoods provided a better estimate of activation overlap than using OA, suggesting that our method is better at aligning task activation across individuals. Notably, this was possible in the absence of spatial regularization as used in (Nenning et al., 2017), and thus spatially remote but functionally close voxels in the embedding space could be the nearest neighbors. Previous studies have suggested that the functional connectivity at rest and task are highly similar and might share an underlying functional architecture (Cole et al., 2014; Elliott et al., 2019; O'Connor et al., 2017). Resting-state networks have been shown to predict neural activity evoked by cognitive tasks (Cole et al., 2016; Tavor et al, 2016), and embeddings of rs-fMRI have also been used as functional features for cortical alignment and improved group-level task activation analysis (Nenning et al., 2017). It is plausible, therefore, that a common high-dimensional embedding space established with rs-fMRI could provide a general way to address a whole range of questions regarding the general functional architecture of the human brain.

### 4.3 Age prediction and lifespan analysis reveal common connectome trajectories

One advantage of aligning individuals in a common representative space is that it can overcome problems of anatomical differences in different populations (e.g. development, aging, disease, and species). In particular, an optimal alignment method can minimize the noise in the identification of biological relevant features and lead to more accurate prediction. In this work, we applied our approach to a lifespan sample with a large age range (6–85 years). JE achieved a moderate accuracy in age prediction (r2 = 0.6450, MAE = 10.79 years) that was significantly higher than the previously established embedding alignment using OA (r2 = 0.5950, MAE = 11.44 years). In prior studies, brain-age prediction using only functional features have shown comparable predictions in developmental cohorts and adult lifespan samples (r2 = 0.38–0.57) (Dosenbach et al., 2010; Li et al., 2018; Nielsen et al., 2019; Qin et al., 2015; Vergun et al., 2013; Wang et al., 2012). It's worth noting that the age prediction using structural or multi-modal features had better prediction in large developmental cohorts (age 5–18, r2 = 0.71, MAE = 1.41 years) and adult lifespan samples (age 18–90, r2 = 0.61, MAE = 3.51 years) (Cole, 2019; Zhao et al., 2019). Overall, therefore, our results are broadly aligned with previous studies of age-related neurocognitive changes. The ability to capture age related changes in the common embedding space generated using JE suggests that this method may be useful in optimizing functional alignment and so facilitate attempts to identify within participant changes that may be important biomarkers of health and disease.

Regression in the common space revealed highly significant trajectories across the lifespan, and particularly quadratic relationships between age and embedding coefficients were observed. Those trajectories exhibited a large overlap with the embedding component per se, suggesting that each embedding dimension reflects a unique feature of brain development. Previous lifespan studies showed that resting-state connectivity decreased within and increased between networks (Betzel et al., 2014), revealed region-specific trajectories of different order characterizing homotopic resting-state connectivity over age (Zuo et al., 2010), and a shift from a local to a more distributed connectivity structure was observed between child- and adulthood (Fair et al., 2009, 2008). Our results illustrated that the range of the second component, capturing the distinction between integration of visual networks, showed a steady decline over age. It was demonstrated that somatosensory and visual regions mature before higher-order association areas (Gogtay et al., 2004), and a decrease in range can be interpreted as an increased integration of visual areas into the global connectivity structure. We also found that both components 1 and 3 increased in range over the first half of lifespan with a subsequent decline towards old age. Notably both these components are linked to complex thought. Component 1 is argued to reflect the dissociation between transmodal and unimodal cortex that supports abstract representations (Margulies et al., 2016; Mesulam, 1998), while Component 3 is similar to the dissociation between task-positive and task-negative regions that is often seen during resting compared to particularly active task conditions (Duncan, 2010; Raichle et al., 2001). Moving forward, the JE common space may help determine the unique neurocognitive trajectories that emerge over the course of the lifespan.

### 4.4 Functional characterizations across individuals in a common space

Finally, it has recently become possible to use multimodal features to improve the inter-individual alignment using both anatomical features (e.g. cortical folding, myelin) and functional features including resting-state functional connectivity, task activation, and retinotopic mapping etc. (Nenning et al., 2017; Paquola et al., 2019; Robinson et al., 2018, 2014). Similar approaches have been applied to align neural signals in the functional domain (e.g. hyperalignment, shared response model, blueprints and semantic mapping etc.) (e.g. hyperalignment, shared response model, blueprints and semantic mapping etc.) (Chen et al., 2015; Cohen et al., 2017; Haxby et al., 2011; Huth et al., 2012; Kriegeskorte and Kievit, 2013; Mars et al., 2018). Such methods map individual connectomes to a common space without topological constraints (Dubois and Adolphs, 2016; Langs et al., 2015). These common spaces are normally constructed to represent the neural response patterns of stimulus, semantic categories, gradients of connectivity topography or shared connectivity profiles (Chen et al., 2015; Guntupalli et al., 2016; Haak et al., 2018; Huntenburg et al., 2017; Huth et al., 2012). One advantage of these common space models is that they enable the uncoupling of function from structure, which can allow comparisons to be made efficiently even when anatomical structures are different (Langs et al., 2016, 2015; Xu et al., 2019). Although these methods all show promise, the identification of the best way to determine common representations with respect to individual differences in the common space remains an ongoing challenge. In the current study, we jointly extract the matched components that simultaneously preserve individual features (Fig. S10). Our results demonstrated that this approach optimized the commonalities of embedding profiles between individuals, and at the same time, achieved better discrimination of individuals from each other. In addition, the JE alignment in a lifespan sample showed an improvement of age prediction. In our recent work, we have demonstrated that JE can also be applied to align functional connectivity across species (Xu et al., 2019). This, we suggest JE as a framework of mapping individual characterizations in a functional common space.

With the growing size of fMRI datasets, it is worth noting that a single joint similarity matrix containing all individuals can be computationally infeasible. This drawback can be circumvented by pairwise concatenating joint similarity matrices for alignment of sequential data, such as lung images with respiratory motion (Baumgartner et al., 2013). However, the alignment of individual functional embeddings is sensitive to subtle differences in the intrinsic structure of each dataset (Li et al., 2019; Wang et al., 2015), rendering potential bias by the initial individual selection. In the current JE approach, we propose to chain the individual connectivity via the group average connectivity structure instead of iteratively jointly embed two individuals. This procedure mutually regularizes the individual embedding representations by the group average and vice versa, resulting in a common space spanned by the group average. However, using a group average connectome as the reference might limit the extent to which subtle individual-specific components can be established. Further improvement could be made by a more optimal choice and construction of a reference space which facilitates the alignment of less common modes of variation, e.g. a group average after functional alignment as the reference common space. Note that JE and OA are both functional alignments in a dimensional space which quantifies functional organization and establishes comparability across individuals without spatial constraints. In response, without anatomical correspondence, the functional space alone may render difficulties in determining the structural basis of functional difference, assessing the commonalities across a group, and accounting for regionally varied confounds of the BOLD response. The investigations of structure-function relationships may need a combination of functional- and anatomy-based alignment in future studies.

### 4.5 Conclusion

In this work, we proposed joint embedding as a general framework to find comparable embedding representations across datasets. We established that joint embedding had better alignment than standard approaches, providing a set of components that have higher within and across individual similarity, while also increasing the ability to identify participants by capturing individual differences. We demonstrated that joint embedding increases task activation overlap across individuals. Importantly, it facilitates the characterization of life span changes in the functional connectivity structure, establishing common trajectories of change, corresponding with more accurate age prediction than orthonormal alignment. Overall, this demonstrates that joint embedding simultaneously captures individual representations in a common functional space, that will help understand both normal and abnormal changes in neural organization.

## CRediT authorship contribution statement

**Karl-Heinz Nenning:** Conceptualization, Formal analysis, Methodology, Software, Visualization, Writing - original draft, Writing - review & editing. **Ting Xu:** Conceptualization, Formal analysis, Methodology, Resources, Writing - original draft, Writing - review & editing. **Ernst Schwartz:** Methodology, Writing - review & editing. **Jesus Arroyo:** Methodology, Writing - review & editing. **Adelheid Woehrer:** Funding acquisition, Writing - review & editing. **Alexandre R. Franco:** Writing - review & editing. **Joshua T. Vogelstein:** Methodology, Writing - review & editing. **Daniel S. Margulies:** Writing - review & editing. **Hesheng Liu:** Resources, Writing - review & editing. **Jonathan Smallwood:** Writing - review & editing. **Michael P. Milham:** Conceptualization, Funding acquisition, Resources, Writing - review & editing. **Georg Langs:** Methodology, Conceptualization, Funding acquisition, Resources, Writing - review & editing.

## Declaration of Competing Interest

None.

## Appendix Supplementary materials

Image, application 1

Image, video 2

Image, video 3
