# Supplementary material for: Joint embedding: A scalable alignment to compare individuals in a connectivity space
Source: Neuroimage. 2020 Nov 15;222:117232. doi: 10.1016/j.neuroimage.2020.117232 (PMC7779372; doi:10.1016/j.neuroimage.2020.117232)
Supplement: Supplementary file 1 [file mmc1.pdf]

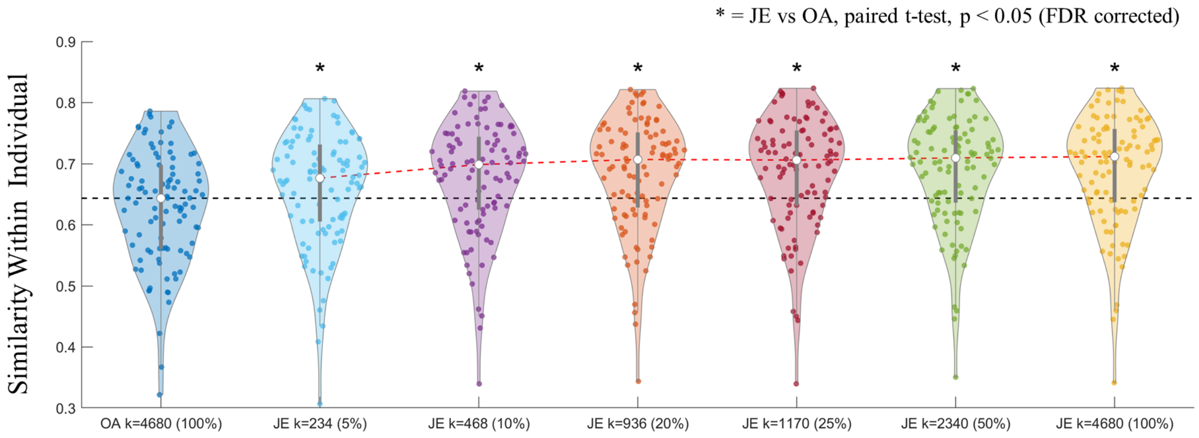

Figure S1. We evaluated the impact of a varying number of k targets, randomly selected across the cortex, from sparsely sampled (5% of all vertices) to densely sampled (100% of all vertices). The similarity of embedding component profiles between two resting-state sessions within each individual was obtained under each sampling condition (5% to 100%). We conducted a paired t-test between orthonormal alignment (OA) and each of the joint embeddings (JE) with varying k targets. Compared to OA, JE with different sampling k provided consistently better similarity, even with a sparse sampling at 5% of cortical vertices.

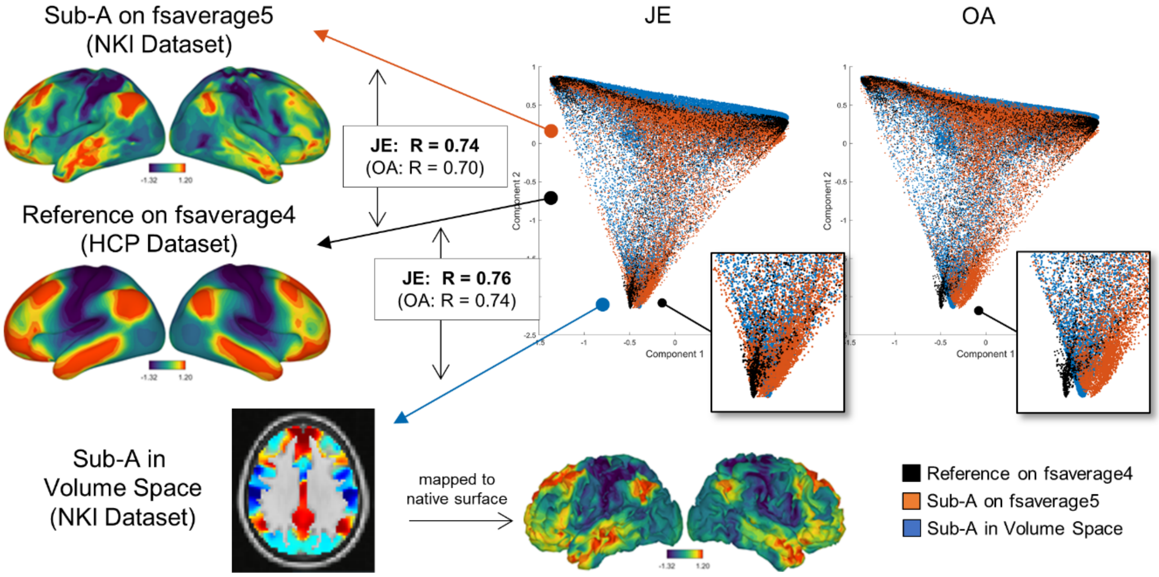

Figure S2. Establishing a common embedding space between a reference in fsaverage4 (black) and one individual in fsaverage5 (orange) and MNI volumetric space (blue). For the joint embedding procedure 400 ROIs (Schaefer et al., 2018; 7 networks parcellation) were used as targets. The average embedding coefficients of those ROIs were used for the matching with JE and OA. Joint embedding provides a more accurate alignment to the reference space.

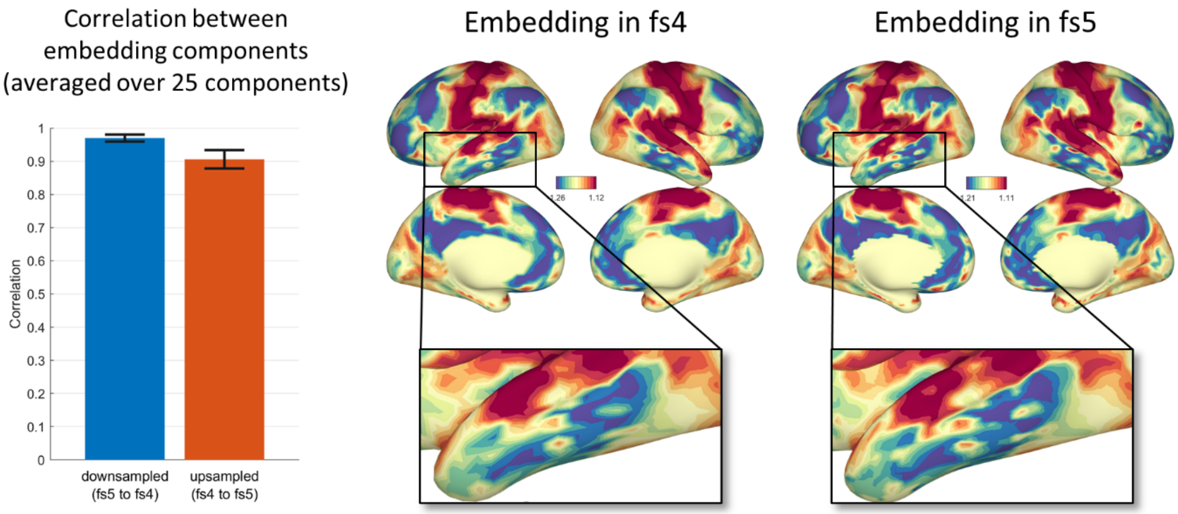

Figure S3. Comparison between diffusion map embeddings calculated in fsaverage4 and fsaverage5. The spatial similarity of the gradient profile by upsampling/downsampling the components to the other surface revealed high spatial correlation across components (mean correlation > 0.9)

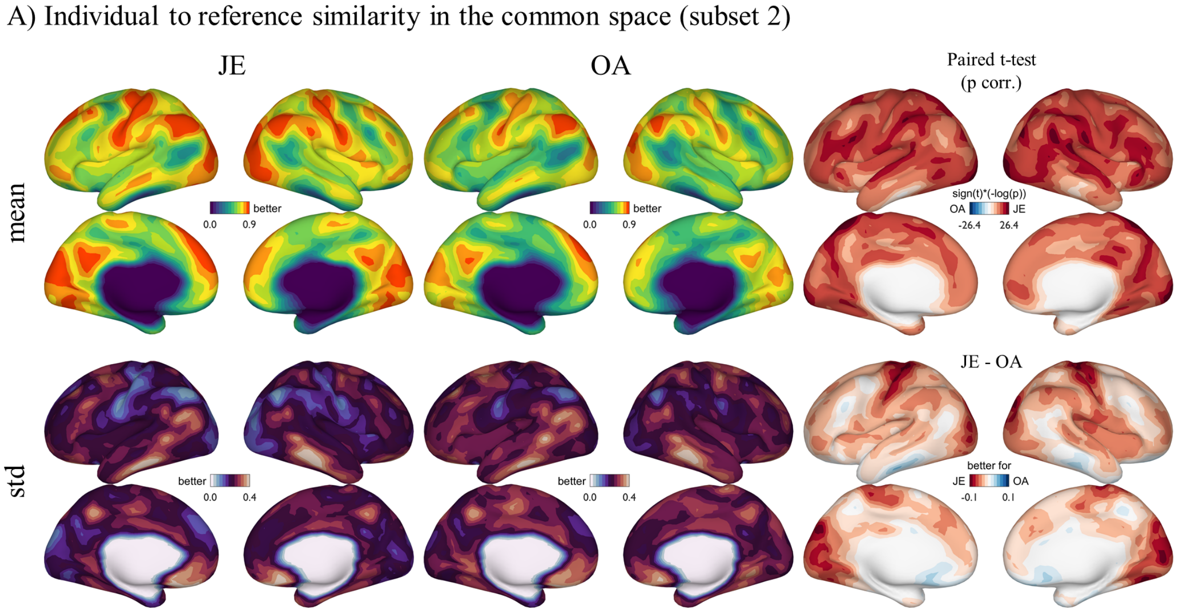

Figure S4. Mean and standard deviation of similarity in embedding component profiles between individuals and reference for subset 2. Results for subset 1 are illustrated in Figure 2.

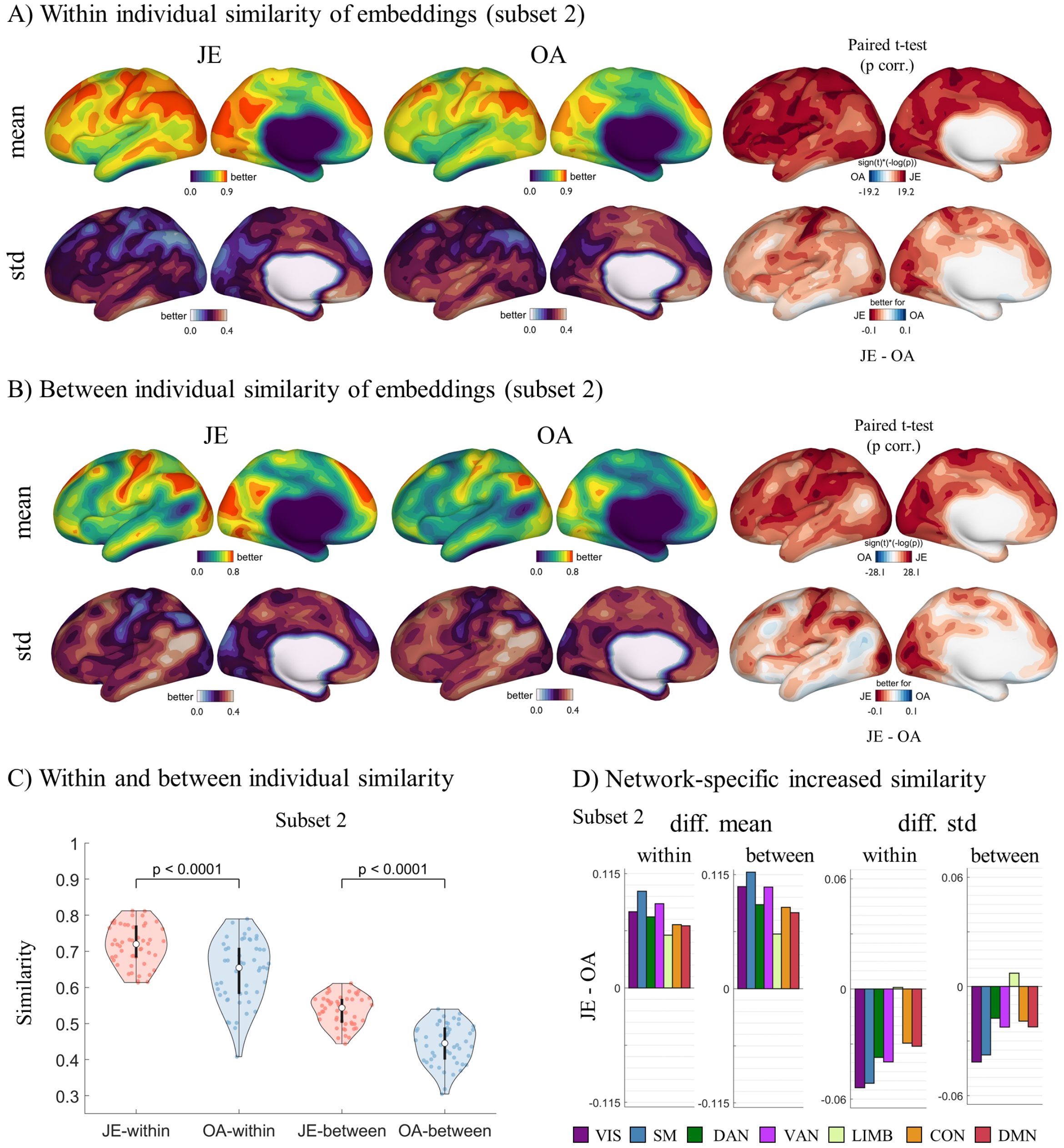

Figure S5. Within and between individual similarity of embedding component profiles for subset 2. A) JE shows increased within individual similarity and reduced variation compared to OA. B) JE shows increased between individual similarity and reduced variation compared to OA. C) A paired t-test reveals significantly higher within and between individuals similarity for JE compared to OA (within: $t(49)=14.26$, $p<0.0001$; between: $t(49)=29.63$, $p<0.0001$). D) Compared to OA, both within and between individual similarities of JE component profiles are higher across networks, whereas the variations are smaller. Results for subset 1 are shown in Figure 3.

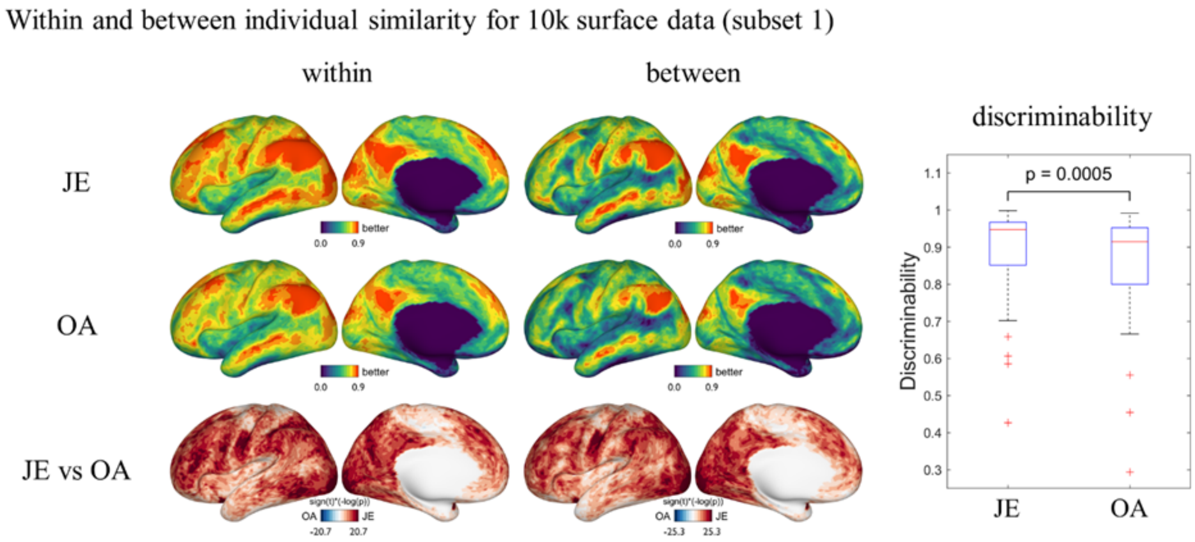

Figure S6. Compared to OA, JE improves the within and between individual matching of embedding components and enables establishing a more coherent connectivity space for data on the 10k_fs_LR surface, which provides a higher discriminability.

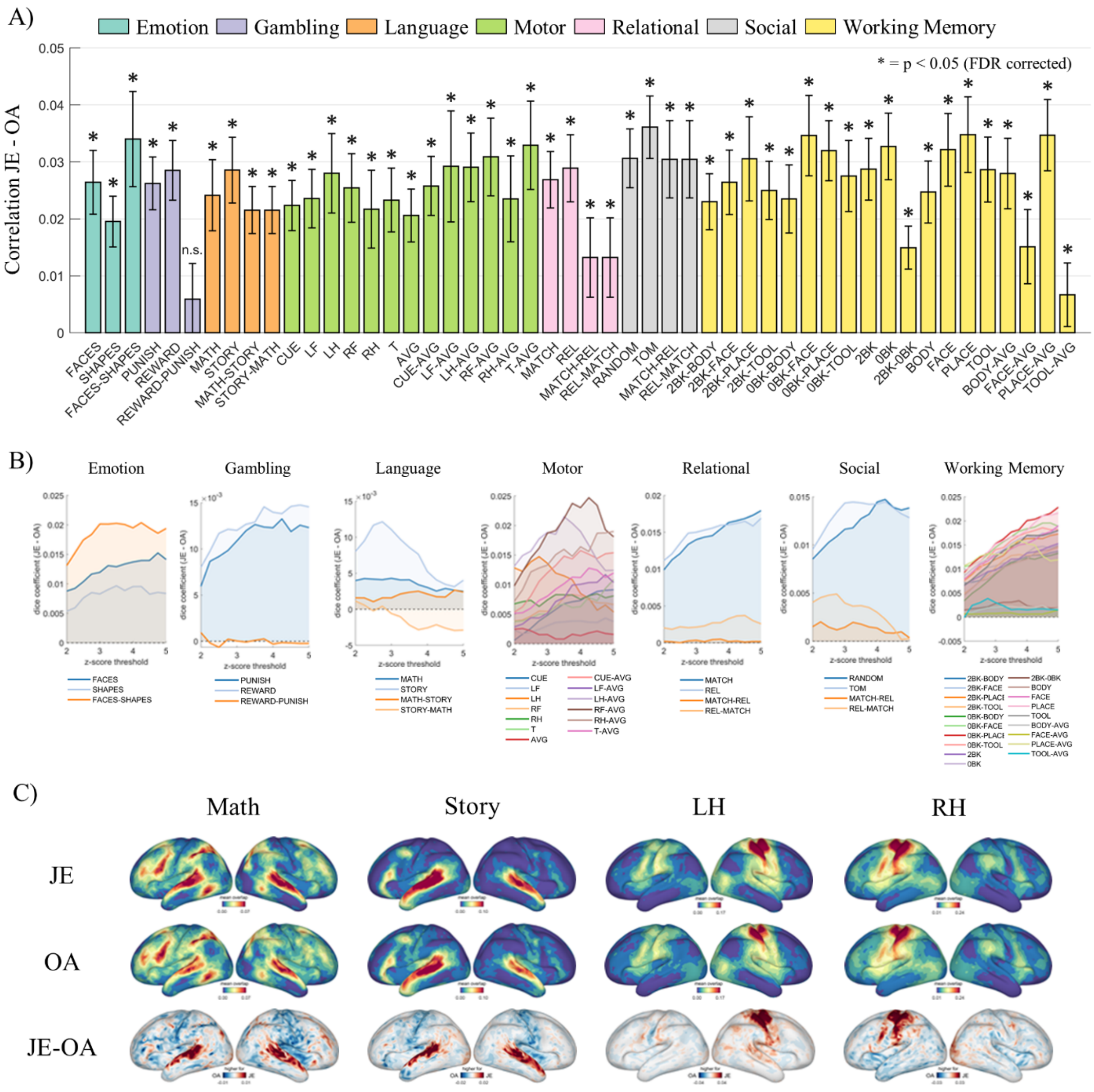

Figure S7. Higher overlap of task-activation across participants in the JE common space compared to OA in subset 2. A) For JE compared to OA, a significantly higher correlation between actual and predicted z-scores is observed in 49 of 50 task-contrasts (paired t-test for 50 individuals in subset, $p < 0.05$ FDR corrected). B) The averaged difference (JE-OA) of pairwise Dice coefficients between thresholded z-maps across individuals at various thresholds. C) The averaged thresholded task-activation maps (z-score > 3.1) show a higher overlap for JE in task-active regions.

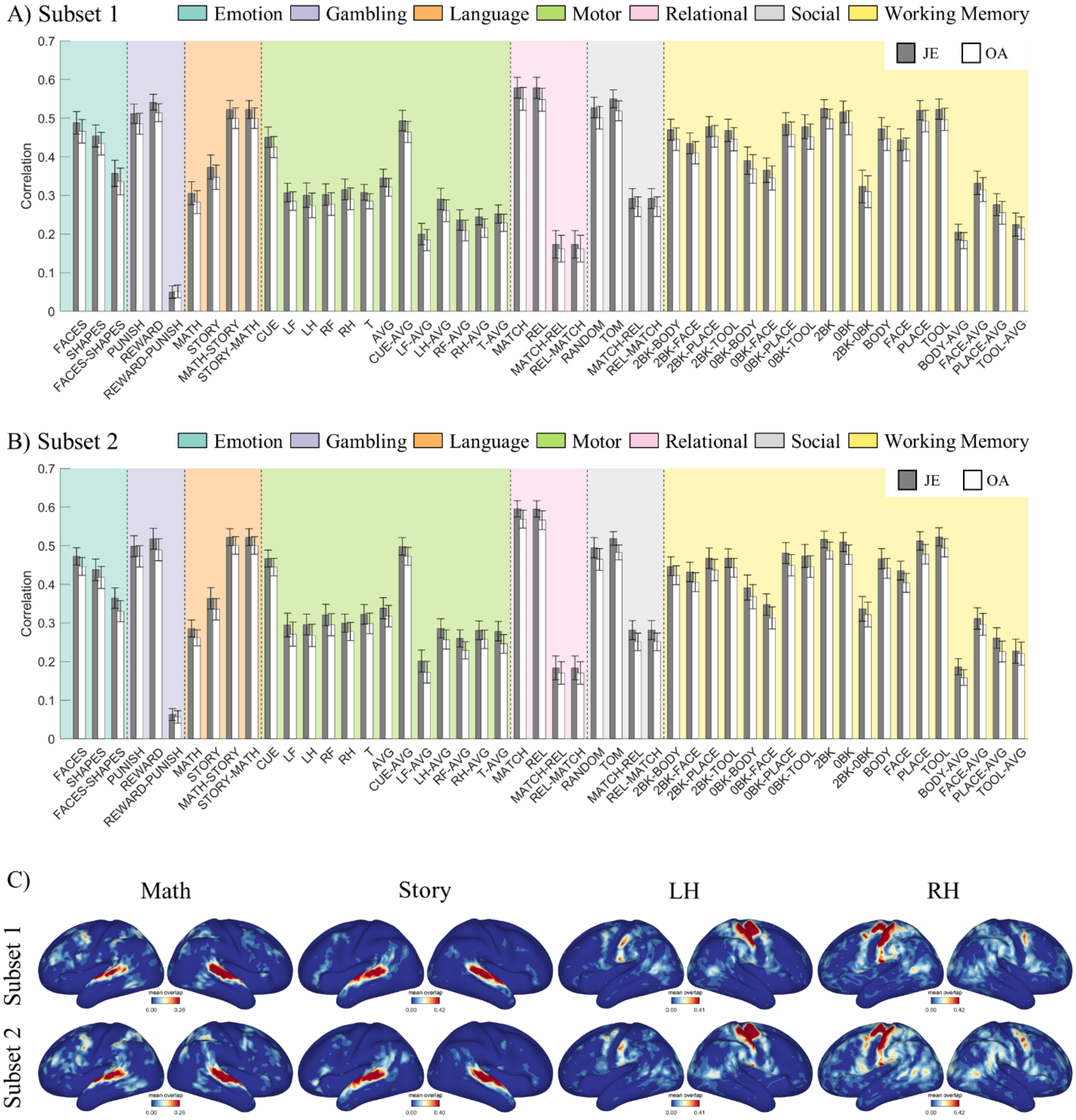

Figure S8. Correlation between actual and predicted z-score maps. A) Subset 1. B) Subset 2. C) Task-activation overlap at z-score > 3.1 for anatomical alignment.

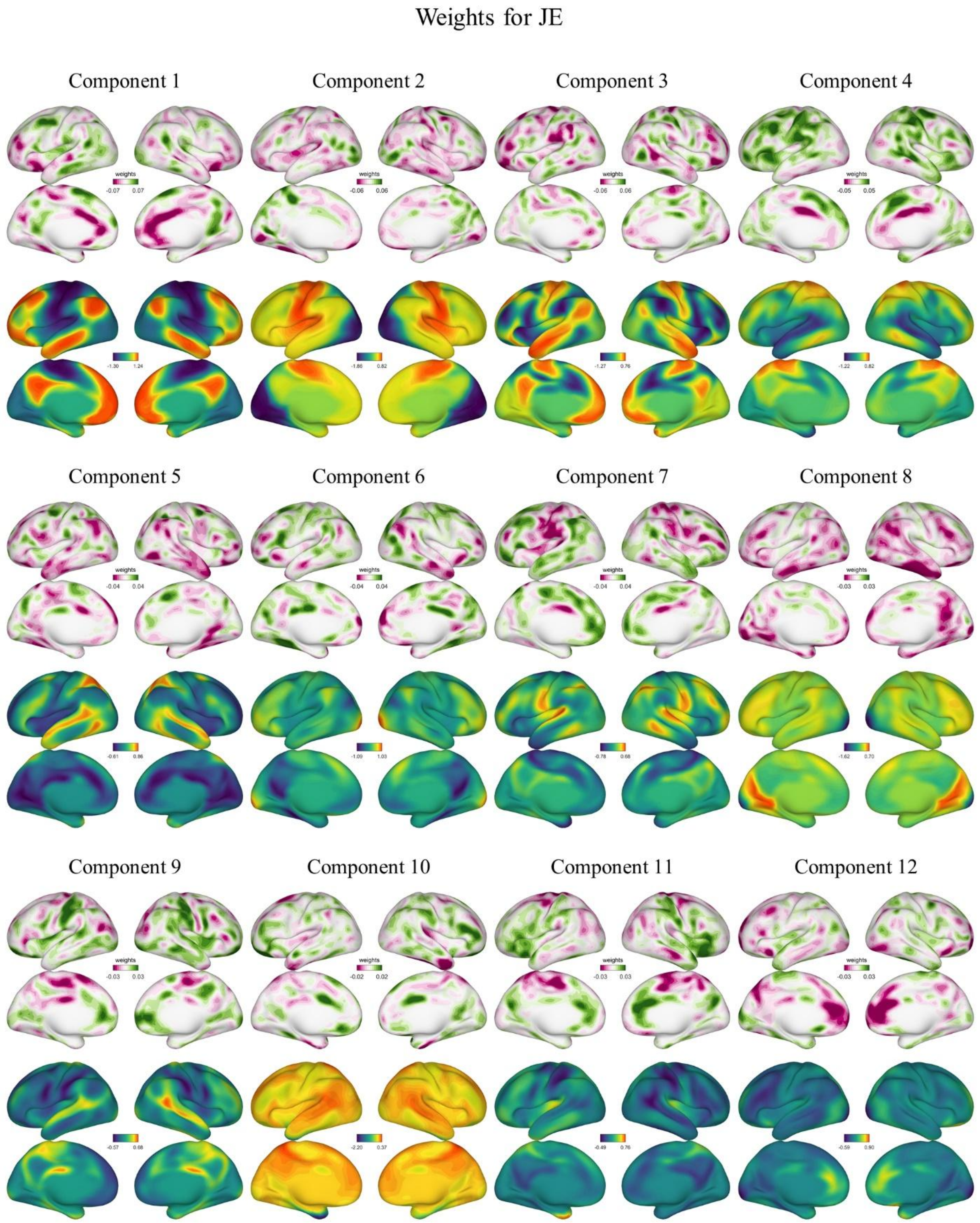

Figure S9. The contribution weights of JE components in age prediction of lifespan sample.

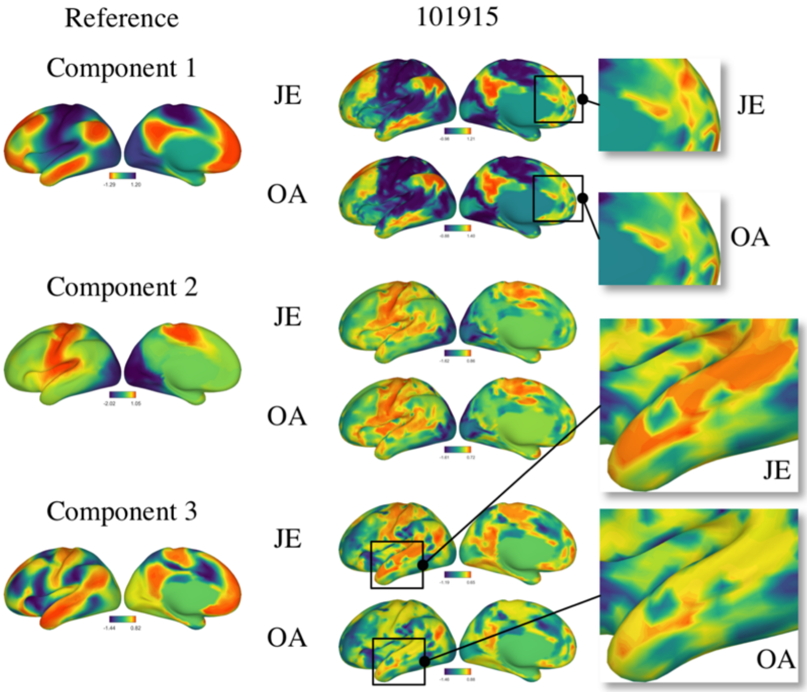

Figure S10. JE preserves and emphasizes individual-specific topological features as compared to OA.

References

Schaefer, A., Kong, R., Gordon, E.M., Laumann, T. O., Zuo, X. N., Holmes, A. J., Eickhoff, S. B., Yeo, B.T., 2018. Local-Global Parcellation of the Human Cerebral Cortex from Intrinsic Functional Connectivity MRI. Cereb Cortex.28(9):3095-3114. doi:10.1093/cercor/bhx179
